# Increased susceptibility of CD4+ T cells from elderly individuals to HIV-1 infection and apoptosis is associated with reduced CD4 and enhanced CXCR4 and FAS surface expression levels

**DOI:** 10.1186/s12977-015-0213-1

**Published:** 2015-10-09

**Authors:** Anke Heigele, Simone Joas, Kerstin Regensburger, Frank Kirchhoff

**Affiliations:** Institute of Molecular Virology, Ulm University Medical Center, 89081 Ulm, Germany

**Keywords:** HIV, Aging, T cell subsets, CXCR4, FAS, Apoptosis

## Abstract

**Background:**

Elderly HIV-1 infected individuals progress to AIDS more frequently and rapidly than people becoming infected at a young age. To identify possible reasons for these differences in clinical progression, we performed comprehensive phenotypic analyses of CD4+ T cells from uninfected young and elderly individuals, and examined their susceptibility to HIV-1 infection and programmed death.

**Results:**

Peripheral blood mononuclear cells (PBMCs) from older people contain an increased percentage of central memory and Th17 CD4+ T cells that are main target cells of HIV-1 and strongly reduced proportions of naïve T cells that are poorly susceptible to HIV-1. Unstimulated T cells from elderly individuals expressed higher levels of activation markers, death receptors, and the viral CXCR4 co-receptor than those from young individuals but responded poorly to stimulation. CD4+ T cells from older individuals were highly susceptible to CXCR4- and CCR5-tropic HIV-1 infection but produced significantly lower quantities of infectious virus than cells from young individuals because they were highly prone to apoptosis and thus presumably had a very short life span. The increased susceptibility of T cells from the elderly to HIV-1 infection correlated directly with CXCR4 and inversely with CD4 expression. The levels of apoptosis correlated with the cell surface expression of FAS but not with the expression of programmed death receptor 1 (PD1) or tumor necrosis factor-related apoptosis-inducing ligand (TRAIL).

**Conclusions:**

Increased levels of activated and highly susceptible HIV-1 target cells, reduced CD4 and enhanced CXCR4 cell surface expression, together with the high susceptibility to FAS-induced programmed cell death may contribute to the rapid CD4+ T cell depletion and accelerated clinical course of infection in elderly HIV-1-infected individuals.

**Electronic supplementary material:**

The online version of this article (doi:10.1186/s12977-015-0213-1) contains supplementary material, which is available to authorized users.

## Background

Aging and HIV-1 infection affect one another and share many characteristics: both are associated with diminished T cell functionality, low production of naïve T cells, loss of regenerative capacity, an accumulation of aging T cells, reduced memory T cell populations, and lower numbers of properly functioning CD8+ cytotoxic T cells (CTLs) [1–4]. Furthermore, many non-AIDS but age-related illnesses, such as hepatic, pulmonary, cardiovascular and renal disease, as well as diabetes mellitus, dementia and arthritis, are substantially more frequent in HIV-1-infected individuals than in age-matched uninfected people [5–7]. Notably, this accelerated aging in virally infected individuals continues to be a problem under highly effective antiretroviral therapy (ART), most likely because even in the absence of detectable viral loads the levels of inflammation remain higher than in healthy uninfected individuals [7, 8].

Aging and HIV-1 infection also have synergistic effects because progression to AIDS occurs substantially faster in elderly infected individuals. Adjusted for general aging effects on mortality, the median survival for those who became HIV-1 infected at ages 25–34 was 11 years, as compared to 4.4 years in those who were infected at 65 years or older [9]. Although elderly HIV-infected individuals progress to AIDS more rapidly than younger people, they usually achieve lower viral loads, possibly due to better adherence to treatment [10]. Possible reasons for the frequent and accelerated disease progression in elderly HIV-infected patients are an age-associated decrease in thymic function [1], replicative senescence of the immune system associated with accelerated telomere shortening [11], and lower CD4 cell counts at baseline [10, 12]. Other factors proposed to contribute to the greater risk of disease progression in elderly HIV-infected patients are increased expression of the CCR5 HIV-1 co-receptor [13], reduced production of IL-2 and its receptor that may promote immunosenescence [14], and reduced CD8+ cytotoxic T cell function [9]. However, the strongest predictors of progression to AIDS in HIV-infected humans of all ages are increased levels of immune activation and apoptosis [5, 15, 16]. Chronic inflammation increases the T cell turnover rates and thus the exhaustion of the regenerative capacity of the immune system associated with AIDS [17, 18].

It is conceivable that age is an important factor in AIDS progression because the regenerative capacity of people becoming HIV-1 infected at an older age is a priori reduced so that they already start with a handicap. However, the immune systems of elderly and younger individuals may also react differently to HIV-1 infection. In fact, recent data suggest that the levels of T cell activation, inflammation and apoptosis are particularly high in older HIV-infected patients [7, 19]. The reasons for these age-dependent differences are currently poorly understood and further studies seem highly warranted since they may provide new insights into the mechanisms underlying the sustained inflammation that drives progression to AIDS and thus ultimately help to optimize antiretroviral therapy in HIV-1-infected individuals. To identify possible reasons for the increased rates and accelerated kinetics of AIDS progression in elderly individuals, we analyzed which properties may render CD4+ T cells from the elderly particularly susceptible to HIV-1 infection and depletion.

## Results

### Young and elderly differ in CD4+ T cell subsets that are targets for HIV-1 infection

**Fig. 1:**
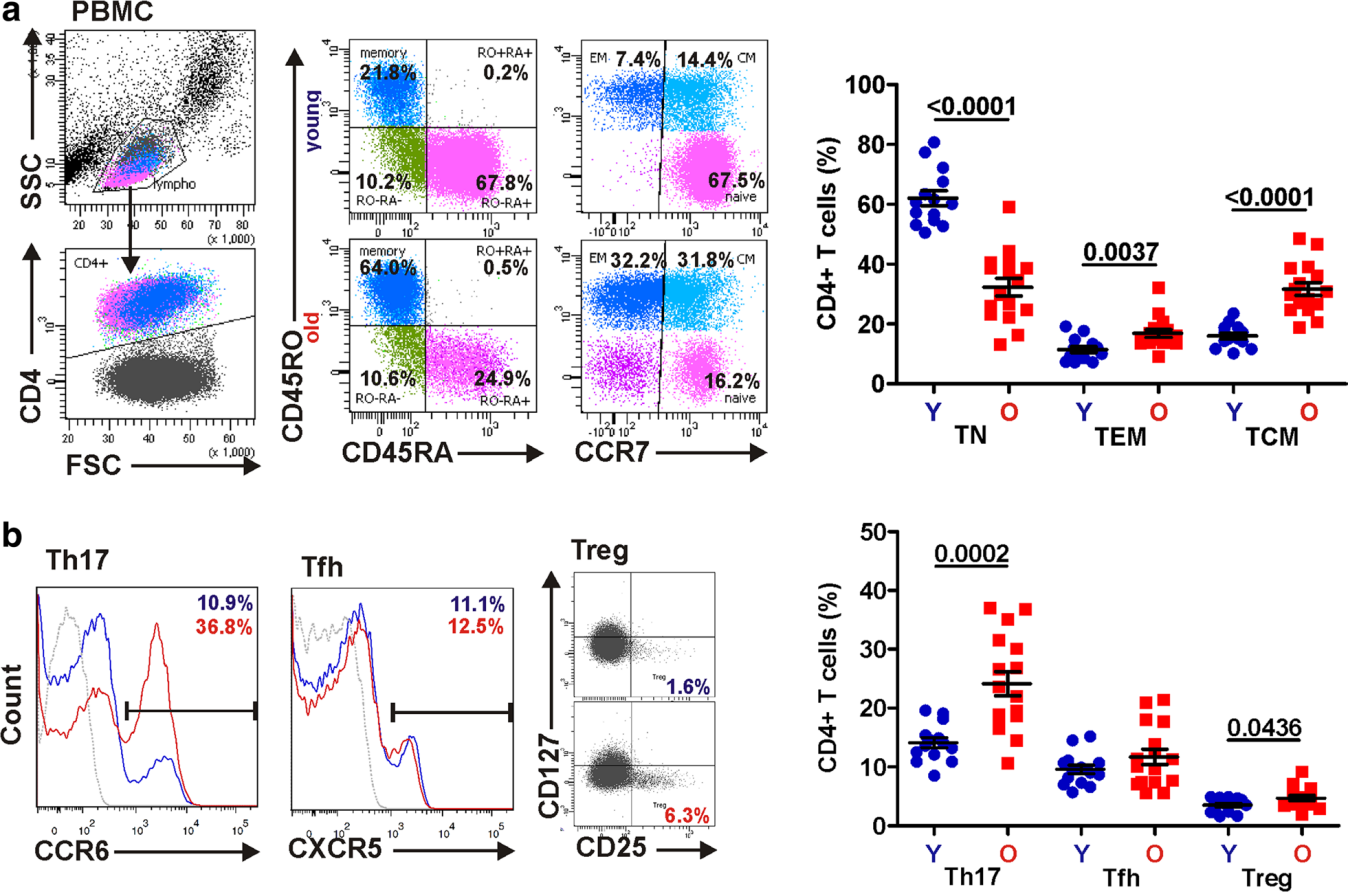
Differences in CD4+ T cell subsets between young and elderly individuals. **a** (*left*) Gating strategy for flow cytometry analysis and representative primary data to determine the percentages of naïve (*TN*), effector memory (*TEM*) and central memory (*TCM*) cells within the CD4+ T cell population from young (*Y*, n = 14) and elderly (*O*, n = 16) healthy blood donors. (*right*) Comparison of percentages of CD4+ naïve, effector and central memory T cells from young and old donors. **b** (*left*) Representative primary data and (*right*) statistical evaluations of the percentage of Th17, Tfh and Treg cells within the CD4+ T cell population. PBMCs isolated by lymphocyte separation medium were kept in supplemented RPMI without further stimulation and analyzed by FACS after overnight incubation. *Grey lines* in this and in other figures represent isotype controls

To define possible differences in the composition and functionality of different CD4+ T cell subsets, we performed phenotypic analyses of uninfected PBMCs from young (range 18–25 years, 22.1 ± 2.2; n = 14) and elderly (range 50–86 years, 62.6 ± 9.1; n = 16) donors. In agreement with published data [20, 21], unstimulated PBMCs from older people contained a strongly reduced proportion of naïve T cells (TN, CD45RA+ CCR7+; 32.3 ± 2.9 % vs. 62.0 ± 2.5 %; p < 0.0001) and increased percentages of effector (TEM, CD45RO+ CCR7−; 16.8 ± 1.3 % vs. 11.5 ± 1.0 %; p = 0.0037) and central memory (TCM, CD45RO+ CCR7+; 31.6 ± 2.1 % vs. 16.1 ± 1.0 %; p < 0.0001) T cells compared to young individuals (Fig. 1a). We also noted a population of CD4+ CD45RA+ CCR7− “terminally differentiated” effector memory (TEMRA) T cells in the elderly that was missing in PBMCs obtained from young people (Fig. 1a). Furthermore, the percentage of CCR6+ Th17 cells (24.2 ± 2.0 % vs. 14.1 ± 0.9 %; p = 0.0002) and of CD25high+ CD127− regulatory CD4+ T cells (Treg, 4.7 ± 0.4 % vs. 3.5 ± 0.3 %; p = 0.0436) was significantly higher in elderly individuals (Fig. 1b). Finally, we observed a slightly higher frequency of CXCR5+ T cells that are reminiscent of follicular helper CD4+ T cells (Tfh) which regulate antigen-specific B cell immunity and may represent a relevant compartment for HIV-1 replication [22] in the elderly (Fig. 1b).

**Fig. 2:**
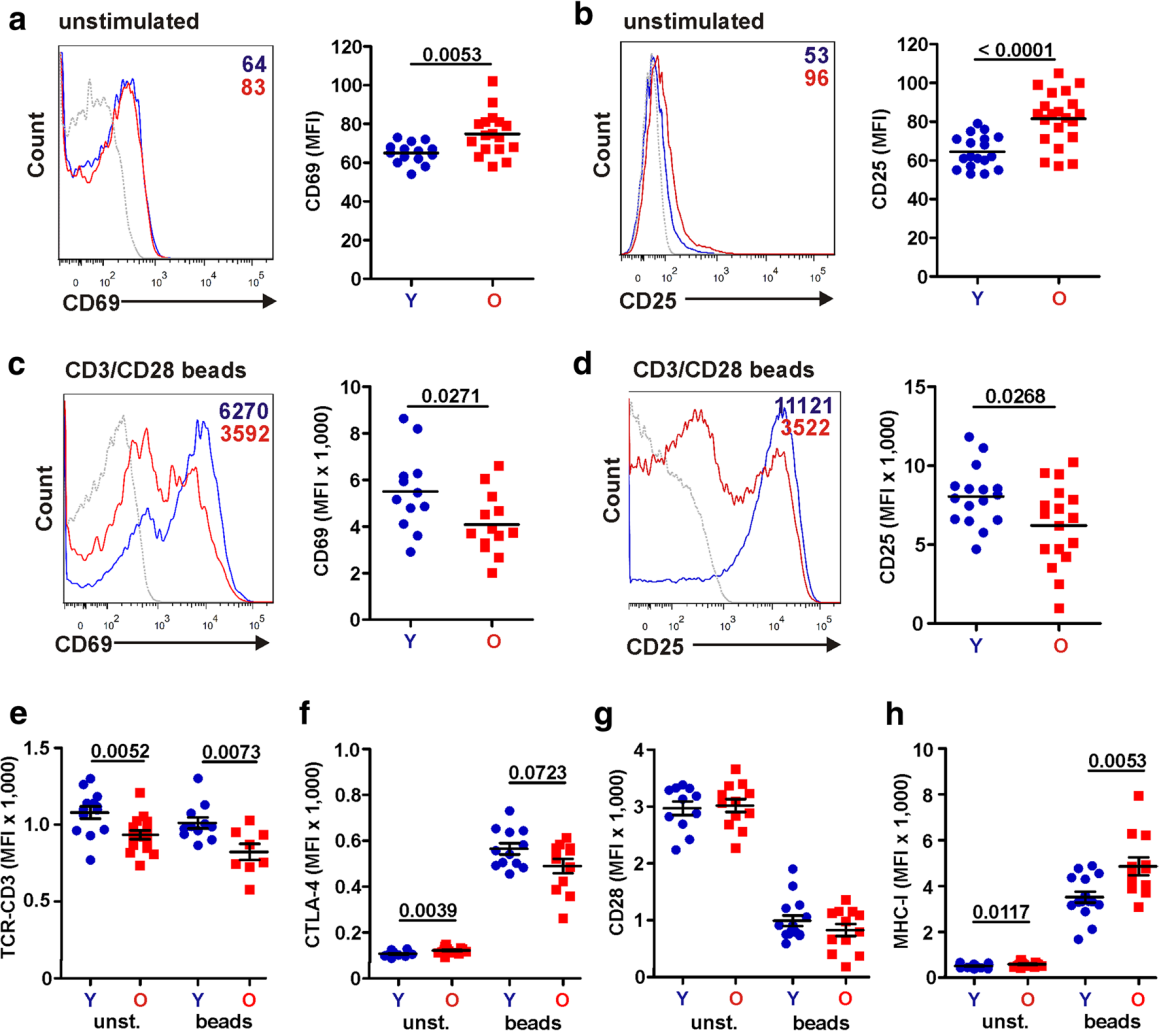
CD4+ T cells from elderly individuals respond poorly to TCR-CD3 stimulation and express increased levels of MHC-I. **a**–**d** Representative primary data and statistical evaluation of the expression levels of **a**, **c** CD69 (early activation marker) and **b**, **d** CD25 (late activation marker) on unstimulated (**a**, **b**) or CD3/CD28 bead stimulated (**c**, **d**) CD4+ T cells from young (*Y*) and elderly (*O*) blood donors. **e**–**h** Expression levels of **e** TCR-CD3, **f** CTLA-4, **g** CD28 and **h** MHC-I on unstimulated or CD3/CD28 bead stimulated CD4+ T cells from young and old donors

To determine the basal activation phenotype and responsiveness to stimulation of CD4+ T cells from young and elderly individuals, we either left them untreated and analysed them 1 day after isolation, or treated them with CD3/CD28 beads that mimic activation by antigen-presenting cells [23] and examined them by flow cytometric analysis 3 days later. The results showed that unstimulated CD4+ T cells from elderly individuals express significantly higher basal levels of activation markers CD69 and CD25 than those from young individuals (Fig. 2a, b). However, T cells from elderly individuals were less responsive and expressed lower levels of these activation markers after stimulation (Fig. 2c, d). Lower levels of TCR-CD3 cell surface expression (934 ± 29 vs. 1079 ± 40, p = 0.0052; Fig. 2e; values give mean fluorescence intensities (MFIs) of TCR-CD3 expression ± SEM) and increased basal expression levels of the inhibitory cytotoxic T-lymphocyte-associated protein 4 (CTLA-4) (121.8 ± 3.3 vs. 107.9 ± 2.9, p = 0.0039; Fig. 2f) may contribute to this reduced responsiveness of CD4+ T cells from elderly individuals. In comparison, the cell surface expression levels of the CD28 co-stimulatory factor did not differ significantly between young and elderly individuals (Fig. 2g). Stimulation with CD3/CD28 beads induced a strong increase in CTLA-4 expression particularly in cells from young individuals (Fig. 2f) and a drastic decrease in CD28 expression (Fig. 2g). Expression of CTLA-4 on stimulated CD4+ T cells correlated significantly with expression of CD25 (R^2^ = 0.8834; p < 0.0001, data not shown). In agreement with an increased state of activation, CD4+ T cells from the elderly expressed higher levels of class I MHC (MHC-I) molecules both before (583 ± 21 vs. 506 ± 20; p = 0.0117) and after (4853 ± 390 vs. 3512 ± 239; p = 0.0053) stimulation (Fig. 2h). Altogether, these analyses show that CD4+ T cells from the elderly show increased basal levels of activation but respond less well to stimulation via the T cell receptor (TCR) complex than T cells derived from young individuals.

### CD4+ T cells from elderly individuals are highly susceptible to HIV-1 infection

**Fig. 3:**
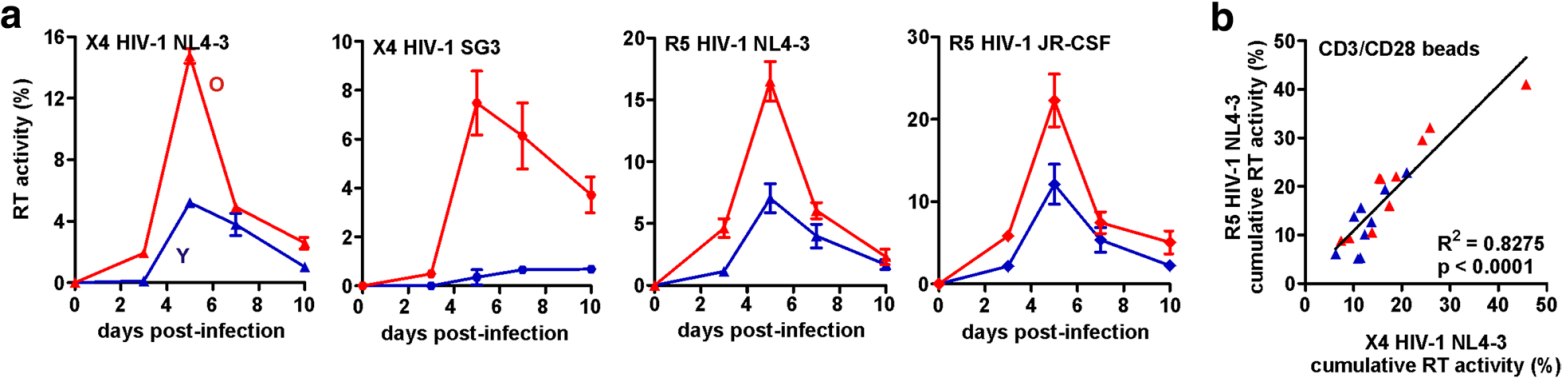
Age-dependent differences in HIV-1 replication. **a** Replication of wild type X4 and R5 HIV-1 strains in PBMCs from young (*Y*, *blue*) and elderly (*O*, *red*) donors upon stimulation with CD3/CD28 beads. Shown are average values (±SEM) from three infections with each HIV-1 molecular clone. The results are representative for the infection of 19 donors performed. **b** Correlation of the cumulative RT production (relative to an RT standard 100 %) by PBMCs infected with X4 or R5 HIV-1 NL4-3 strains after stimulation with CD3/CD28 beads. Each symbol represents the result obtained for one individual PBMC donor from the young (*blue*) or elderly (*red*) groups

The flow cytometric analyses described above revealed phenotypic differences between CD4+ T cells from young and elderly individuals that might affect their ability to promote HIV-1 replication. To further examine this, we stimulated PBMCs derived from young or elderly people with CD3/CD28 beads, exposed them to the wild type CXCR4(X4)-tropic HIV-1 NL4-3 and SG3 strains, or a CCR5(R5)-tropic derivative of NL4-3 [24] and the R5-tropic JR-CSF strain, and determined the efficiency of viral replication by measuring the reverse transcriptase (RT) activity in the culture supernatants. CD3/CD28 beads mimic the primary and co-stimulatory signal of T cell activation by antigen-presenting cells (APCs) and thus provide a physiologically relevant mechanism of activating T cells [23]. We found that the levels of X4 and R5 HIV-1 replication were typically significantly higher in PBMC cultures from older individuals (Fig. 3a). The levels of X4 and R5 HIV-1 replication varied substantially in PBMCs from different donors but correlated significantly with one another (Fig. 3b) suggesting that they are not determined by the viral CXCR4 and CCR5 co-receptors.

To further examine the impact of donor age on the susceptibility of CD4+ T cells to HIV-1 infection and replication, we used X4 and R5 HIV-1 NL4-3 reporter viruses co-expressing the enhanced version of the green fluorescent protein (eGFP) and Nef via an internal ribosome entry site (IRES) [25, 26]. These HIV-1 reporter constructs express all viral proteins and replicate in infected PBMC cultures. Importantly, they readily allow determining both the efficiency of virus production, as well as the frequency and phenotype of HIV-1-infected (GFP+) and uninfected (GFP−) cells in FACS-based assays.

**Fig. 4:**
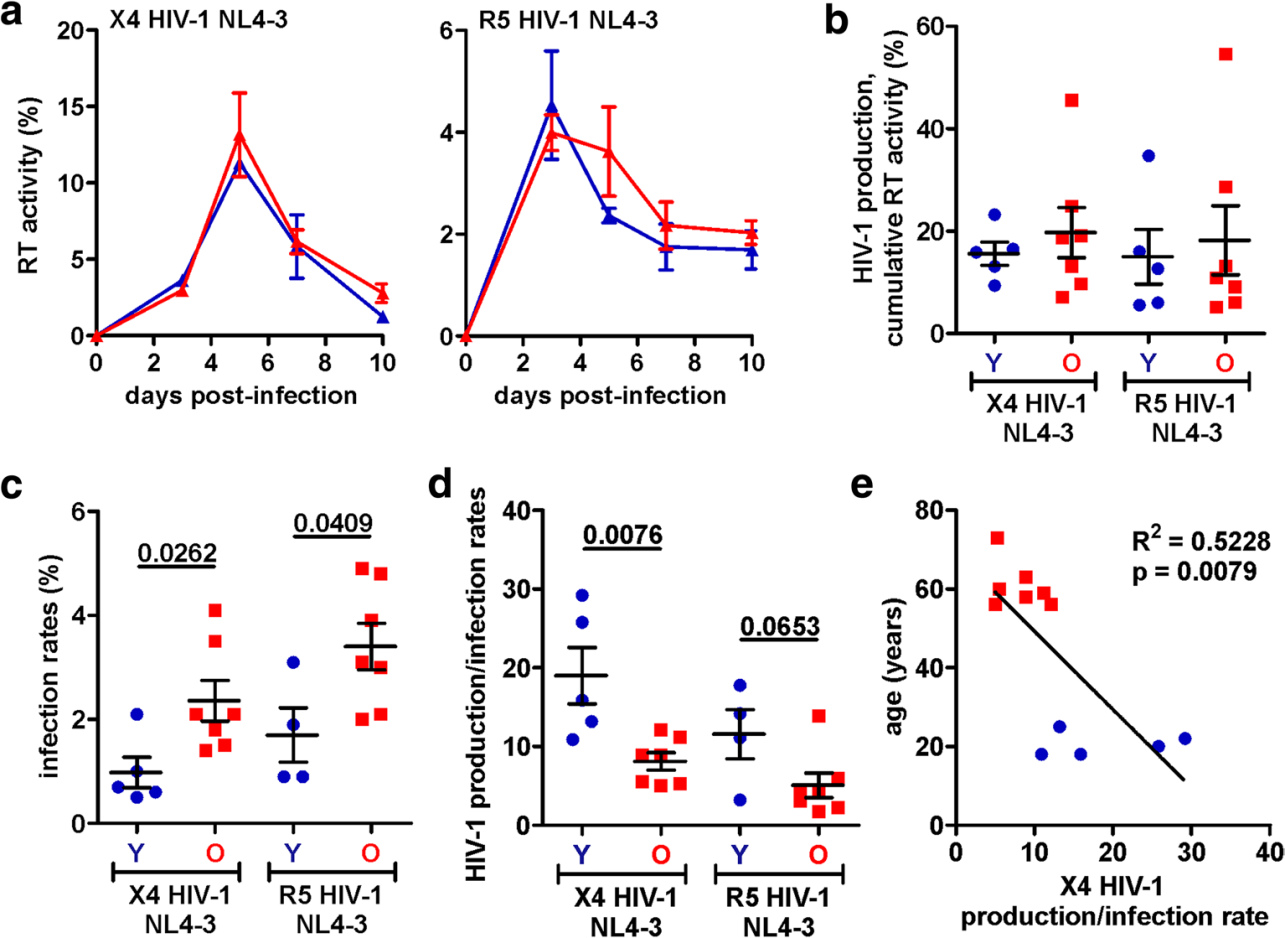
Susceptibility of CD4+ T cells from young and elderly individuals to HIV-1 infection and replication. **a** Replication kinetics of NL4-3 X4 or R5 GFP reporter virus constructs in PBMCs. Infections were carried out after stimulation with CD3/CD28 beads using virus stocks containing 1 ng p24 antigen. Shown are mean values (±SEM) of six infections of PBMC cultures from young (*blue*) or elderly (*red*) donors. **b** Cumulative levels of RT production at days 5, 7, and 10 post-infection for each infected PBMC culture. **c** Average numbers of HIV-1-infected GFP+ cells detected in PBMC cultures at days 3, 5, and 7 post-infection. **d** Levels of HIV-1 RT activity in the cell culture supernatants (cumulative 5, 7, 10 dpi) divided by the average numbers of virally infected (GFP+) cells (values from 3, 5, 7 dpi). **e** Correlation between the age of the PBMC donors and the normalized quantity of HIV-1 production. Each symbol represents the result obtained for one individual PBMC donor

On average, X4 and R5 HIV-1 NL4-3 IRES-eGFP constructs replicated with similar efficiencies in CD3/CD28 bead-stimulated PBMC cultures from young and elderly individuals (Fig. 4a, b). Flow cytometric analysis revealed, however, that the frequency of HIV-1-infected (GFP+) cells was significantly higher (X4 HIV-1, 2.36 ± 0.38 vs. 0.98 ± 0.29, p = 0.0262; R5 HIV-1, 3.40 ± 0.45 vs. 1.70 ± 0.52, p = 0.0409) in PBMC cultures from the elderly (Fig. 4c). Calculation of the levels of virus production for a normalized quantity of HIV-1-infected (GFP+) cells showed that cells from the elderly produced less virus than cells obtained from young individuals (X4 HIV-1, 8.1 ± 1.1 vs. 19.0 ± 3.6, p = 0.0076; R5 HIV-1, 5.1 ± 1.6 vs. 12.8 ± 2.1, p = 0.0653; Fig. 4d). Thus, the levels of virus production per percentage of infected cell correlated significantly (p = 0.0079) with the age of the PBMC donors (Fig. 4e). Infectious virus production by the HIV-1 IRES-eGFP constructs is moderately delayed compared to the parental wild type HIV-1 constructs [27]. Thus, a shortened life span of the infected cell may affect replication of the reporter viruses more severely than the corresponding wild type HIV-1 constructs. This most likely explains why only the latter replicated substantially more efficiently in CD3/CD28 bead-stimulated PBMCs from the elderly (Fig. 3a). We also examined age-dependent differences in HIV-1 replication after stimulation with phyto-hemagglutinin (PHA). However, under these experimental conditions the levels of cell death in PBMC cultures from the elderly were frequently too high for meaningful analyses (data not shown).

### CD4+ T cells from elderly individuals show high levels of apoptosis

**Fig. 5:**
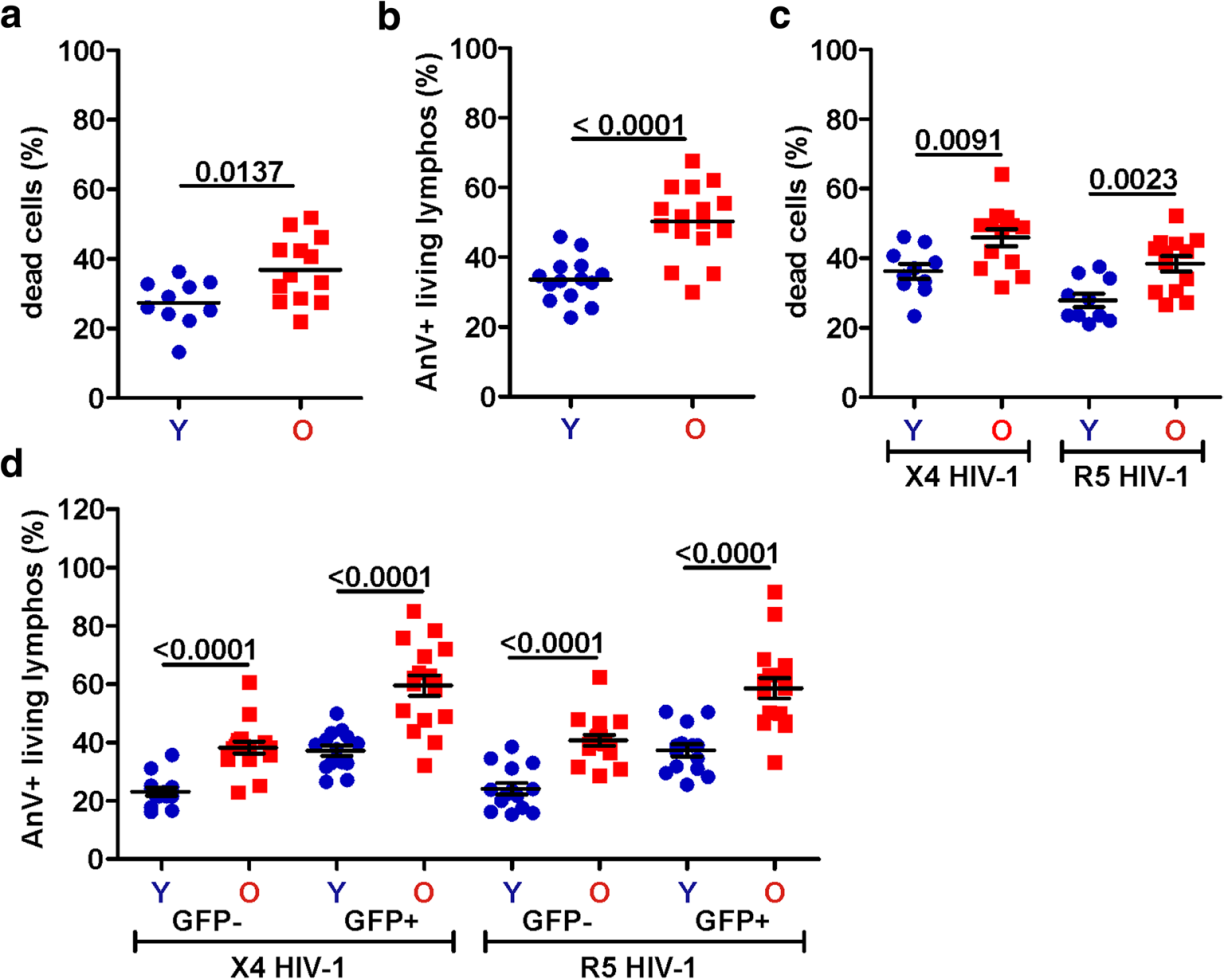
Increased levels of apoptosis in lymphocytes from elderly donors. **a** Death rates and **b** levels of apoptosis in living lymphocytes in uninfected CD3/CD28 bead stimulated PBMC cultures from young (*Y*) and elderly (*O*) donors. **c** Death rates and **d** levels of apoptosis in (*left*) GFP− and (*right*) HIV-1-infected GFP+ cells in PBMC cultures infected with X4 or R5 GFP reporter HIV-1 NL4-3 strains. Each *symbol* represents the result obtained for one individual PBMC donor

The results suggested that CD4+ T cells from the elderly are highly susceptible to HIV-1 infection but may show reduced virus production rates due to high levels of apoptosis that might be associated with a shortened average life span of virally infected T cells compared to young individuals. To address this, we measured the levels of apoptosis in PBMC cultures that were either left uninfected or infected with X4 or R5 NL4–3 IRES-eGFP viruses (an example of the gating scheme is shown in Additional file 1: Figure S1). Even in the absence of HIV-1 infection the frequencies of dead cells (Fig. 5a) and apoptotic lymphocytes (Fig. 5b) were significantly increased in PBMCs derived from elderly individuals (36.9 ± 2.6 vs. 27.4 ± 2.1, p = 0.0137 and 50.3 ± 2.4 vs. 33.6 ± 1.7, p < 0.0001, respectively). However, age-dependent differences in the frequencies of dead cells (Fig. 5c) and the levels of apoptosis in the living lymphocyte population were even more significant in PBMC cultures infected with X4 (GFP+ cells, 59.6 ± 2.5 vs. 27.3 ± 1.8, p < 0.0001; Fig. 5d); or R5 HIV-1 strains (GFP+ cells, 58.6 ± 3.5 vs. 37.3 ± 2.1, p < 0.0001; Fig. 5d). The levels of apoptosis in productively HIV-1-infected (GFP+) cells were significantly higher than in the GFP− cell population (p < 0.0001), irrespectively of the viral co-receptor tropism or the age of the PBMC donors (Fig. 5d).

**Fig. 6:**
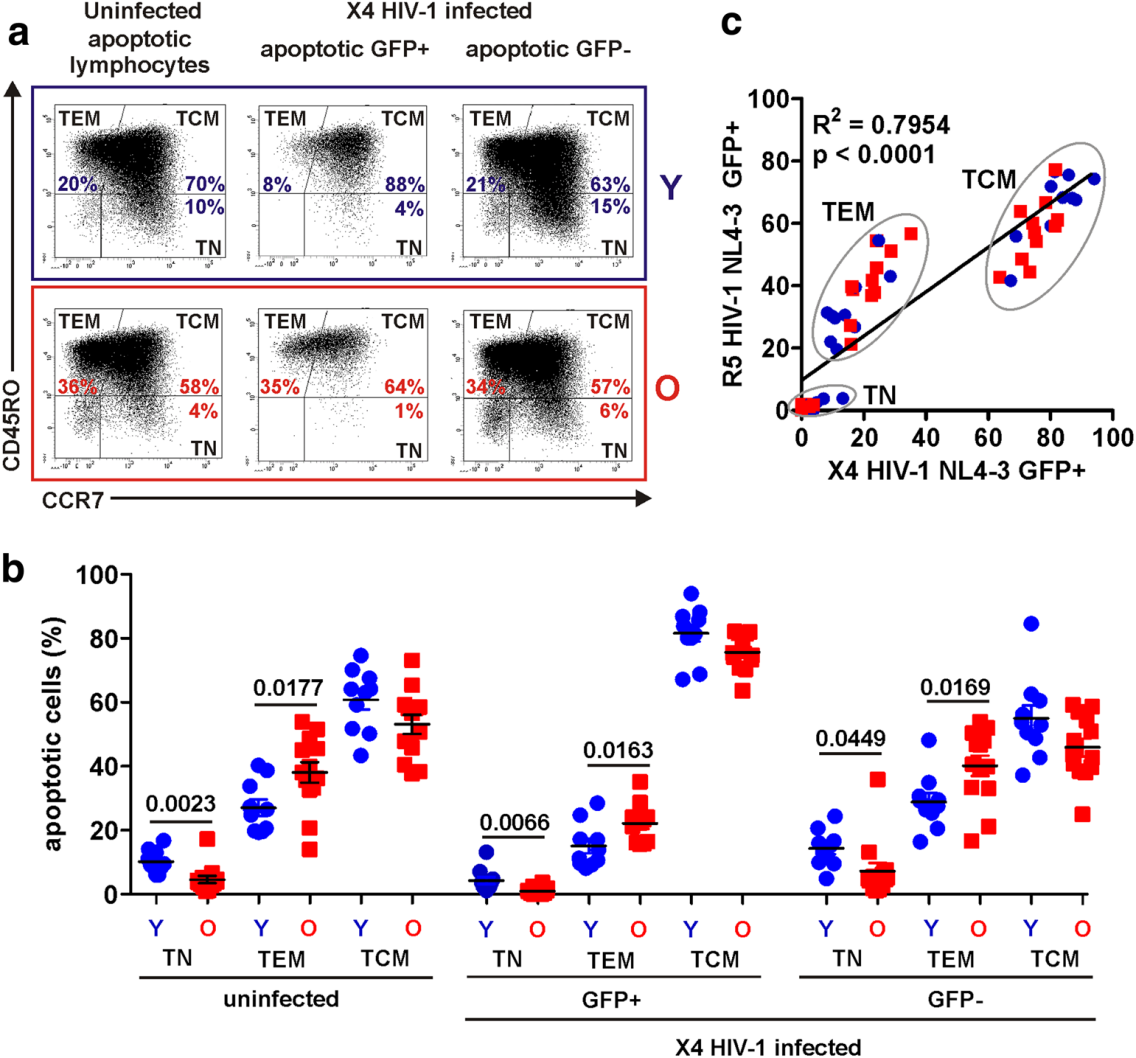
Group-specific differences in HIV-1-infected and uninfected apoptotic T cell populations. **a** Flow cytometric analysis of the apoptotic lymphocyte populations in uninfected PBMC cultures and in the GFP+ and GFP− populations of cultures infected with X4 HIV-1 NL4-3 GFP reporter virus after CD3/CD28 bead stimulation from young (*Y*) and elderly (*O*) blood donors. Within the apoptotic cell population (100 %) the proportions of T cell subsets with a naïve (*TN*), effector memory (*TEM*) or central memory (*TCM*) phenotype were determined. **b** Statistical evaluation of the proportions of the indicated cell subsets within the apoptotic lymphocyte population of uninfected or X4 HIV-1 IRES-eGFP-infected PBMC cultures. **c** Correlation between the indicated apoptotic T cell subsets within X4 and R5 HIV-1 NL4-3 reporter virus infected PBMC cultures

Next, we determined which specific T cell subsets are apoptotic in uninfected and HIV-1-infected CD3/CD28 bead-stimulated PBMC cultures from young and elderly individuals. For this, we examined CD45RO and CCR7 expression (Fig. 6a) of the apoptotic (AnnexinV+) subset of living lymphocytes (Additional file 1: Figure S1). TCM cells generally represented the majority and TN the minority of apoptotic lymphocytes (Fig. 6b). The percentage of TN cells in the apoptotic cell population was increased and that of TEM cells significantly decreased in younger individuals (Fig. 6b), as expected from the overall age-dependent differences in these T cell subsets (Fig. 1a). In agreement with our previous finding that TN cells are largely refractory to X4 HIV-1 infection [28], they were strongly underrepresented in the GFP+ apoptotic lymphocyte population (Fig. 6b). In contrast, the percentage of TCM cells was significantly increased (81.7 ± 2.6 vs. 55.1 ± 4.1, p < 0.0001 and 75.7 ± 1.7 vs. 46.0 ± 2.8, p < 0.0001) in the apoptotic HIV-1-infected (GFP+) population compared to AnnexinV+/GFP− cells in the same culture (Fig. 6b). Altogether, similar results were obtained with the R5 HIV-1 derivative (Fig. 6c). However, the frequency of apoptotic TEM cells was markedly increased and that of TN cells even further decreased in apoptotic R5 HIV-1-infected (GFP+) T lymphocyte populations (Fig. 6c).

### The levels of apoptosis in HIV-infected CD4+ T cells correlate with FAS expression

**Fig. 7:**
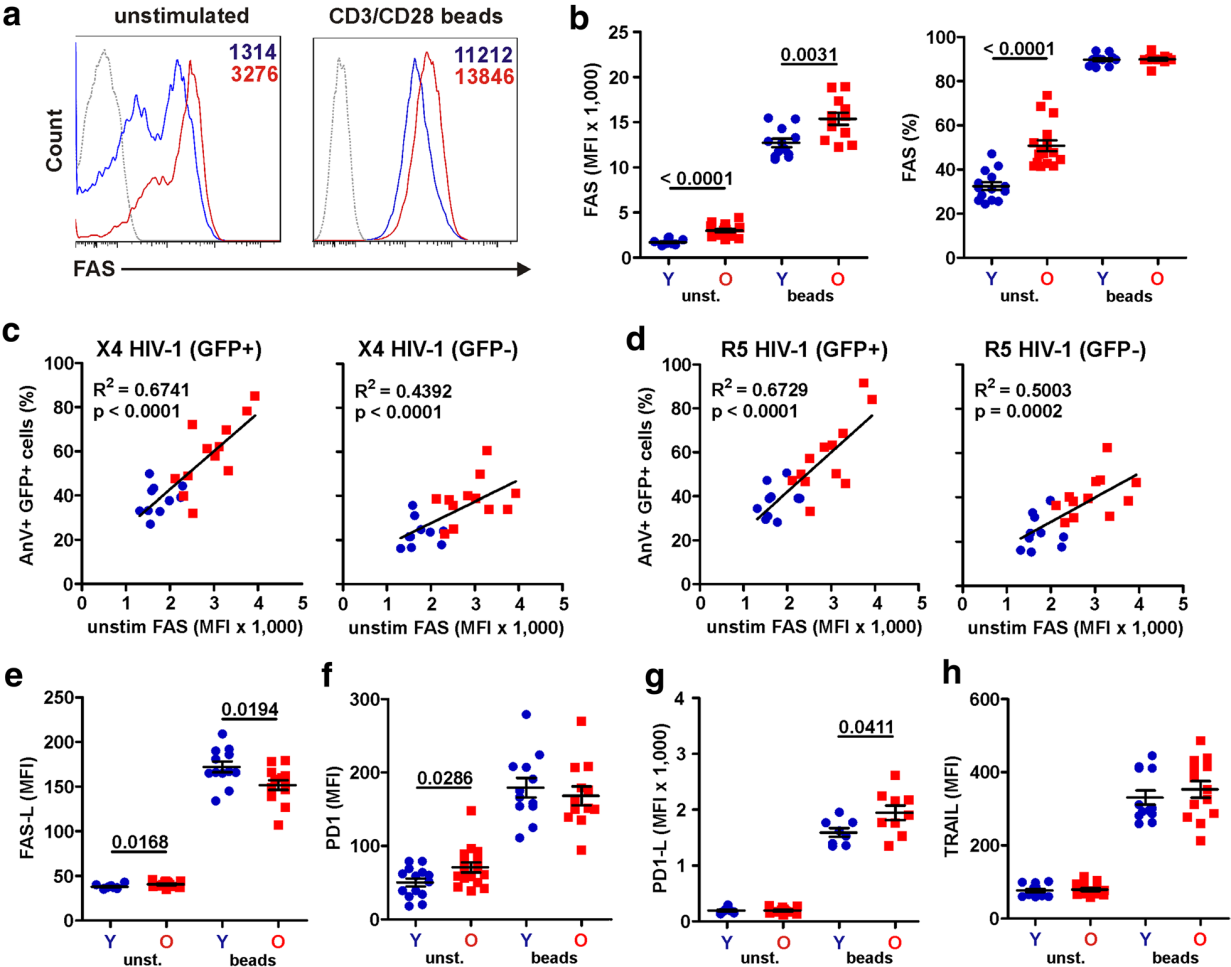
HIV-1 causes higher levels of FAS-dependent apoptosis in T cells from older than from younger individuals. **a** Representative primary data (*left*) and **b** statistical evaluation (*right*) comparing expression levels of FAS on unstimulated and CD3/CD28 bead stimulated CD4+ T cells from young (*Y*, *blue*) and old (*O*, *red*) donors. **c**, **d** Correlation analysis of FAS expression levels on unstimulated CD4+ T cells and apoptosis rates in the GFP+ and GFP− populations of PBMC cultures infected with **c** X4 or **d** R5 HIV-1 NL4-3 IRES-eGFP constructs. **e**–**h** Expression levels of **e** FAS ligand, **f** PD1, **g** PD1 ligand and **h** TRAIL on unstimulated or CD3/CD28 bead stimulated CD4+ T cells from young and elderly donors

To identify correlates of the high levels of apoptosis in HIV-1-infected T cells from the elderly, we examined the expression of various death receptors. CD4+ T cells from elderly people expressed substantially higher levels of FAS compared to those obtained from young individuals particularly prior to stimulation (2995 ± 172 vs. 1697 ± 78, p < 0.0001; Fig. 7a, b). Perhaps most notably, the levels of FAS expression detected prior to CD3/CD28 bead stimulation correlated very well with the rates of apoptosis in X4 and R5 HIV-1-infected (GFP+) CD4+ T cells, whereas the correlation with programmed cell death in the uninfected (GFP-) cell population was less significant (Fig. 7c, d). No significant correlation was observed between the levels of FAS expression prior to and after stimulation and between FAS expression on activated CD4+ T cells and HIV-induced apoptosis (data not shown). In comparison to FAS, the levels of FAS ligand (FAS-L) and programmed death receptor 1 (PD1) in unstimulated CD4+ T cells were only slightly increased in elderly (Fig. 7e, f). Furthermore, the levels of PD1 ligand (PD1-L) and TRAIL cell surface expression did not differ significantly between both age groups (Fig. 7g, h). Stimulation of the cells with CD3/CD28 beads strongly induced expression of all these death receptors (Fig. 7). Only the levels of FAS and PD1-L expression, however, were significantly higher in elderly than in young individuals. Furthermore, the levels of FAS-L, PD1, PD1-L and TRAIL expression did not correlate with the percentages of apoptotic CD4+ T cells (data not shown). Thus, FAS seems to play a key role in the high levels of apoptosis in HIV-1-infected PBMC cultures from older people.

### Inverse correlation between CD4 cell surface expression and virus production

**Fig. 8:**
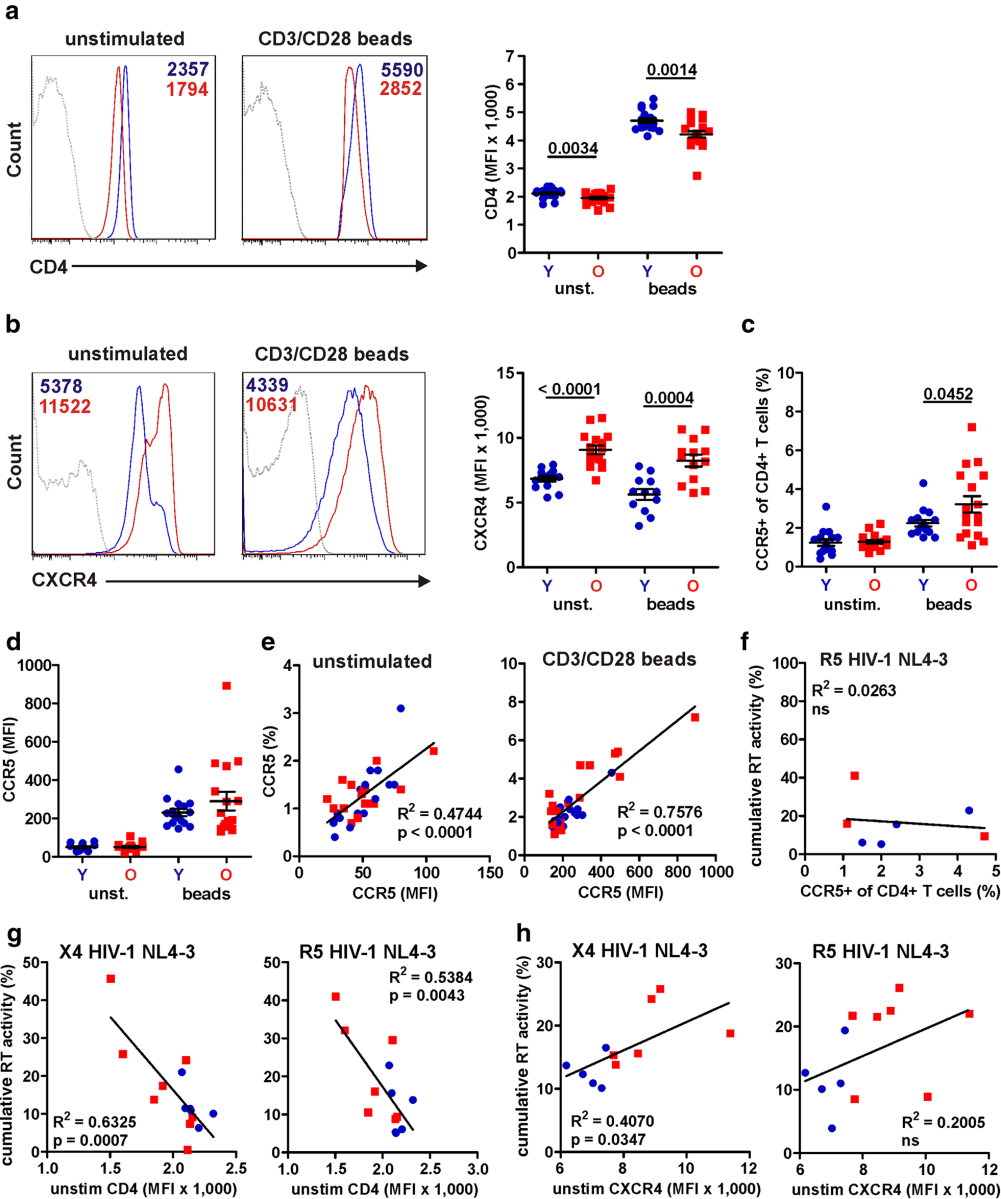
Age-dependent differences in CD4 and CXCR4 expression. **a** Representative primary data (*left*) for unstimulated or CD3/CD28 bead stimulated CD4+ T cells stained for CD4 or **b** CXCR4 expression levels. **a** (*right*) Statistical analysis of CD4 or **b** (*right*) CXCR4 expression levels on unstimulated and stimulated CD4+ T cells from young (*Y*) and elderly (*O*) donors. **c** Percentages and **d** mean fluorescence intensity (*MFI*) of CCR5+ within the total CD4+ T cells population in unstimulated and stimulated PBMC cultures from young (*Y*) and elderly (*O*) donors. **e** Correlation between CCR5 expression levels (MFI) on total CD4+ T cells and percentages of CCR5+ within total CD4+ T cells in unstimulated or stimulated PBMC cultures. **f** Correlation between percentages of CCR5+ within total CD4+ T cells in stimulated PBMC cultures and the cumulative levels of RT production in R5 HIV-1 NL4-3 infected PBMC cultures. Correlation between the **g** CD4 and **h** CXCR4 surface expression levels on unstimulated CD4+ T cells and the cumulative levels of RT production in X4 or R5 wild type HIV-1 NL4-3 infected PBMC cultures

To identify reasons for the high susceptibility of CD4+ T cells from the elderly to HIV-1 infection, we examined the cell surface expression levels of the primary CD4 receptor and the CCR5 and CXCR4 co-receptors, needed for HIV-1 entry. We found that the levels of CD4 expression were significantly lower (unstimulated, 1949 ± 37 vs. 2111 ± 36, p = 0.0034; stimulated, 4217 ± 113 vs. 4782 ± 82, p = 0.0014; Fig. 8a) and those of the CXCR4 co-receptor significantly higher (unstimulated, 9064 ± 330 vs. 6834 ± 200, p < 0.0001; stimulated, 8228 ± 425 vs. 5620 ± 408, p = 0.0004; Fig. 8b) in uninfected PBMC cultures from elderly individuals. The percentage of CCR5+ T cells was also significant higher in stimulated cells from the elderly (Fig. 8c). In comparison the difference in CCR5 co-receptor expression levels failed to reach significance (Fig. 8d) although the frequency of CCR5+ T cells and the MFI correlated with one another (Fig. 8e). Most notably, CCR5 expression did not correlate with the efficiency of R5 (and X4) HIV-1 replication (Fig. 8f and data not shown). In contrast, low levels of CD4 surface expression correlated inversely with replication efficiency of both X4 and R5 HIV-1 (Fig. 8g), whereas high levels of CXCR4 correlated only with increased production of X4 but not R5 HIV-1 (Fig. 8h). Thus, reduced levels of CD4 and high levels of CXCR4 expression may promote efficient HIV-1 replication in older people.

Finally, we examined whether differences in the levels of activation of HIV-1-infected T cells from young and elderly individuals might also affect the efficiency of viral replication. We found that HIV-1-infected (GFP+) T cells expressed substantially higher levels of the early T cell activation marker CD69 than uninfected GFP- cells irrespective of the donor’s age or viral co-receptor tropism (Additional file 1: Figure S2A). In contrast, the levels of CD25 expression did not differ significantly between uninfected and infected T cells (Additional file 1: Figure S2B). However, CD25 expression in the HIV-1-infected population of the elderly was slightly increased compared to the young age group (Additional file 1: Figure S2B). To better assess whether this modest difference in the late state of T cell activation might affect viral LTR activity, we determined the MFIs of eGFP expression in HIV-infected primary T cells. eGFP is expressed together with Nef by the regular HIV-1 LTR promoter and thus represents an indicator of the viral transcriptional activity. However, the MFIs of eGFP expression did not differ significantly between the young and elderly age groups (Additional file 1: Figure S2C). Thus, age-dependent differences in the efficiency of HIV-1 infection are not due to differences in the efficiency of viral gene expression.

## Discussion

In the present study, we identified several differences in the phenotypes and properties of CD4+ T cells from young and elderly people that may play a role in the high susceptibility of older individuals to HIV-induced immunodeficiency [6, 29]. PBMC cultures from the elderly contain high percentages of TCM cells that are primary targets for HIV-1 replication but low percentages of naïve CD4+ T cells, which are relatively resistant to HIV-1 infection [30, 31]. Furthermore, the proportion of CCR6+ Th17 cells, reported to be highly permissive for HIV-1 infection [32] was significantly enhanced in elderly individuals. Finally, we observed a slightly higher frequency of follicular helper CD4+ T cells (Tfh) that regulate antigen-specific B cell immunity and may represent a major compartment for HIV-1 replication [22] in the elderly (Fig. 1b). Thus, an increased age was associated with an increased frequency of CD4+ T cells subsets known to be highly susceptible to HIV infection.

In addition to the age-dependent differences in T cell subsets, T cells from the elderly expressed significantly lower levels of the primary CD4 receptor and higher quantities of the CXCR4 co-receptor of HIV-1 entry compared to T cells from young individuals. These findings are in agreement with published data, reporting an increased frequency of CD4low T cells [33], and significant increases in CXCR4 surface expression [34] in uninfected elderly individuals. At first view, the inverse correlation between CD4 expression and the efficiency of HIV-1 replication may seem striking. Previous cell culture studies have shown, however, that reduced levels of CD4 cell surface expression may facilitate efficient virus release and enhance the infectivity of progeny HIV-1 virions [35–37]. The advantage of low levels of CD4 cell surface expression is perhaps most evident from the fact that HIV-1 uses three of its ten gene products (Env, Vpu and Nef) to down-modulate its primary receptor from the cell surface [27]. Increased levels of CXCR4 expression in T cells from the elderly may also seem surprising since this receptor is predominantly expressed on naive CD4+ T cells [38] that are significantly reduced in the elderly (Fig. 1a). However, increased surface CXCR4 expression in the elderly has been observed previously and may be due to altered ubiquitination of this co-receptor [34]. Enhanced CXCR4 expression might facilitate viral entry and helps to explain why the percentages of X4 HIV-1-infected GFP+ cells were significantly higher in the old age group. Our results thus suggest that low CD4 and high CXCR4 expression both contribute to the high susceptibility of T cells from the elderly to HIV-1 infection.

Our results support the presumption that the overall efficiency of HIV-1 replication depends on a delicate balance between the susceptibility of the CD4+ target T cells to HIV-1 infection and the levels of programmed death that might limit the life span of the infected cells and thus the time frame of virus production. These findings help to explain why HIV-1 may cause more damage in the elderly even at lower viral loads [39]. On the one hand, increased levels of T cell activation and CXCR4 cell surface expression together with reduced CD4 expression levels render CD4+ T cells from the elderly highly susceptible to HIV-1 infection and replication. On the other hand, HIV-1-infected PBMC cultures from the elderly showed very high levels of apoptosis and the short span of infected CD4+ T cells was frequently associated with reduced virus production. Follow-up studies using HIV-1 reporter constructs co-expressing Nef and eGFP confirmed that PBMC cultures from the elderly show particularly higher levels of HIV-1-infected cells but reduced virus production efficiencies on a per cell basis after stimulation with CD3/CD28 beads due to accelerated cell death (Fig. 4). Increased numbers of activated CD4+ T cells that are highly susceptible to HIV-1 infection together with an increased sensitivity of virally infected cells to programmed death might help to explain why older HIV-1-infected patients show a more rapid loss of CD4+ T cells and progression to AIDS than younger individuals [29].

We found a highly significant correlation between the levels of FAS expression on CD4+ T cells prior to stimulation and the levels of apoptosis in HIV-1-infected T cells following stimulation with CD3/CD28 beads. It is known that the frequency of FAS expressing CD4+ T cells [40] and the susceptibility to FAS-induced apoptosis [41] is increased in elderly individuals. Our observation that the levels of FAS but not of TRAIL and FAS-L expression correlate significantly with the levels of apoptosis in HIV-1-infected T cells and are strongly increased in the elderly, suggests that this death receptor plays a relevant role in the high susceptibility of old individuals to HIV-induced immunodeficiency. In agreement to previous studies [42], we detected higher CTLA-4 expression in unstimulated T cells from the elderly. It has been reported that CTLA-4 signaling may result in high CCR5 expression and enhanced susceptibility to viral infection [43]. In the present study, however, the levels of CCR5 cell surface expression did not differ significantly between the groups of the young and the elderly. Upon stimulation with CD3/CD28 beads the CTLA-4 expression levels were even significantly reduced in the elderly (Fig. 2f) and the role of this inhibitory receptor for the pathogenesis of HIV-1 infection in vivo remains to be elucidated. Another factor that was expressed at significantly higher levels in unstimulated CD4+ T cells from the elderly was PD1, which is associated with T cell exhaustion and disease progression [44]. Thus, CD4+ T cells of the elderly show higher basal expression levels of death receptors and activation markers but react poorly to stimulation. In agreement with previous studies [45–47], the levels of FAS were strongly increased in T cells from the elderly. Most importantly, they correlated significantly with the levels of apoptosis in HIV-1-infected CD4+ T cells suggesting that FAS-mediated cell death may play an important role in the high sensitivity of elderly people to HIV-1 induced immune damage.

To approximate the in vivo situation, we used primary CD4+ T cells from young and elderly individuals, exposed them to HIV-1 particles containing regular Env glycoproteins, and performed most stimulations with CD3/CD28 beads. Nonetheless, the relevance of our findings for the difference in the rates of disease progression between young and elderly people remains to be determined. For example, it will be interesting to further examine whether the increased levels of CXCR4 expression in the elderly is associated with an increased frequency of the CCR5 to CXCR4 co-receptor switch that is associated with rapid progression to AIDS in untreated HIV-1-infected individuals [48, 49]. Finally, our observation that high sensitivity of CD4+ T cells to FAS-mediated apoptosis may contribute to rapid disease progression in elderly HIV-infected individual suggests that this death receptor may be a useful target for the prevention of inflammation and CD4+ T cells depletion.

## Conclusions

People becoming HIV-infected at an older age do not only start with a handicap because their immune system has reduced regenerative capacity but also provide a particular susceptible environment for HIV-1 infection and immune damage.

## Methods

### Samples from young and elderly individuals

This study was approved by the Ethics Committee of Ulm University Medical Center. The age range of the young blood donors was 18–25 years (22.1 ± 2.2; n = 14) and of the elderly donors 50–86 years (62.6 ± 9.1; n = 16). Blood was collected from donors, which provided written consent or obtained from the blood donation center, Ulm. To the best of our knowledge, all blood donors were clinically healthy at the time of sampling.

### Isolation and culture of PBMCs

Peripheral blood mononuclear cells (PBMCs) from healthy human donors were isolated using lymphocyte separation medium (Biocoll separating solution, Biochrom) and stimulated for 3 days with human T-activator CD3/CD28 Dynabeads at a bead to cell ratio of 1:1 (Life technologies, 11132D) and 10 ng/ml IL-2 in RPMI1640 medium with 10 % FCS prior to infection. For FACS analysis in unstimulated PBMCs cells were incubated without stimulus in supplemented RPMI overnight.

### T cell subsets and receptor expression

One day post isolation FACS analysis was used to determine the percentages of various CD4+ T cell subsets, like naïve (TN), effector memory (TEM), central memory (TCM), T helper 17 cells (Th17), follicular helper T cells (Tfh) and regulatory T cells (Treg). For this the following antibodies were used: CD4-APC H7 (BD Biosciences, 560158), CD45RA-PE Cy7 (BD Biosciences, 560675), CD45RO-PerCP Cy5.5 (BD Biosciences, 560607), CCR7-APC (BioLegend, 353214), CCR6-PE (BD Biosciences, 559562), CXCR5-PerCP Cy5.5 (BioLegend, 335001), CD127-PE Cy7 (BD Biosciences, 560822), CD25-Horizon V450 (BD Biosciences, 560355). Further, the expression of different receptors on unstimulated as well as stimulated CD4+ T cells was determined using the following antibodies: CD4-APC H7, TCR-CD3-APC H7 (BD Biosciences, 560275), CD28-PerCP Cy5.5 (BD Biosciences, 560685), MHC-I-Horizon V450 (BD Biosciences, 561346), FAS-PE (BD Biosciences, 556641), FAS-L-PE (BioLegend, 306407), PD1-APC (BD Biosciences, 558694), PD1-L-PE Cy7 (BD Biosciences, 558017), TRAIL-PE (BD Biosciences, 550516), CTLA-4-PE (BD Biosciences, 555853), CD69-Horizon V450 (BD Biosciences, 560740), CD25-PE Cy7 (BD Biosciences, 557741), CXCR4-PerCP Cy5.5 (BD Biosciences, 560670) and CCR5-PE Cy7 (BD Biosciences, 557752). Cells were analyzed using the BD FACS Canto II with FACSDiva software.

### Viral constructs

For FACS analysis X4- (env wild type) and R5- (env V3 loop 92th014.12) [24] tropic reporter NL4-3 proviral constructs encoding an internal ribosome entry site (IRES) and the *eGFP* gene upstream of the *nef* gene were used [25, 26]. For wild type replication kinetics proviral constructs of X4-tropic HIV-1 NL4-3 and SG3 as well as R5-tropic HIV-1 NL4-3 and JR-CSF were used.

### Virus stocks

To generate viral stocks, 293T cells were transfected with the proviral constructs as described [26]. The medium was changed after overnight incubation, and virus was harvested 24 h later. For replication kinetics, virus stocks were quantified using a p24 antigen capture assay provided by the NIH AIDS Research and Reference Reagent Program.

### HIV-1 replication in PBMCs

PBMCs were isolated from young and old blood donors and stimulated with CD3/CD28 beads or PHA in the presence of IL-2. Three days post stimulation PBMCs were infected with p24 normalized wild type NL4-3 X4 or R5, SG3 X4, JRCSF R5 viruses for wild type replication kinetics and with NL4-3 X4 or R5 GFP reporter viruses [25, 26] for GFP kinetics. One day post infection the inoculum was removed, cells were washed and fresh medium containing IL-2 was added. Cells were cultured for 10 days and supernatant samples were taken every 2–3 days. The extent of viral replication was determined by reverse transcriptase (RT) assay as described previously [50]. As standard a serial dilution of a HIV-1 NL4-3 virus stock was used and 30 ng/ml p24 was defined as 100 %. In addition FACS analysis were done to determine the percentages of infected cells (GFP+) in the GFP kinetics.

### Apoptosis and activation in infected PBMC cultures

PBMCs were isolated from young and old blood donors and stimulated with CD3/CD28 beads and IL-2. Three days post stimulation PBMCs were infected with NL4-3 X4 or R5 reporter viruses. Two days post infection a second stimulus (beads + IL-2) was added. FACS analysis was done 1 day post second stimulus to determine CD69 expression levels and 2 days post second stimulus to determine CD25 expression levels, apoptosis, apoptotic cell populations, and death rates. For this the following antibodies and dyes were used: CD69-PE Cy7 (BD Biosciences, 557745), CD25-PerCP Cy5.5 (BD Biosciences, 560503), AnnexinV-PE (BD Biosciences, 556421) and fixable viability stain 450 (FVS) (BD Biosciences, 562247). Antibodies used to distinguish TN, TEM and TCM cells in the apoptotic population are mentioned above.

### Statistical analysis

Groups were compared using a two-tailed Student’s t test. The PRISM package version 4.0 (Abacus Concepts, Berkeley, CA, USA) was used for all calculations. In most experiments data from 14 young and 16 elderly blood donors were available for comparison.

## Additional file

10.1186/s12936-015-0927-5 Increased levels of apoptosis in lymphocytes from elderly donors. Gating strategy of flow cytometry analysis of death and apoptosis rates from mock or HIV-1 NL4-3 reporter virus infected GFP+ or GFP− cells. **Figure S2**. Expression of activation markers and viral LTR activity in HIV-1 infected PBMC cultures from young and elderly individuals. (A, B) Representative primary data and statistical evaluations of the expression levels of (A) CD69 and (B) CD25 on X4 or R5 HIV-1 NL4-3 reporter virus infected GFP+ or GFP− cells from young (Y) and elderly (O) blood donors. (C) GFP expression levels in PBMC cultures from young and elderly donors infected with X4 or R5 HIV-1 NL4-3 reporter constructs. Each symbol represents the result obtained for one individual PBMC donor from the young (blue) or elderly (red) groups.

## References

[1] Douek DC, McFarland RD, Keiser PH, Gage EA, Massey JM, Haynes BF (1998). Changes in thymic function with age and during the treatment of HIV infection. Nature.

[2] Naylor K, Li G, Vallejo AN, Lee WW, Koetz K, Bryl E (2005). The influence of age on T-cell generation and TCR diversity. J Immunol..

[3] Weinberger B, Welzl K, Herndler-Brandstetter D, Parson W, Grubeck-Loebenstein B (2009). CD28-CD8+ T cells do not contain unique clonotypes and are therefore dispensable. Immunol Lett.

[4] Effros RB (2004). From Hayflick to Walford: the role of T-cell replicative senescence in human aging. Exp Gerontol..

[5] Appay V, Sauce D (2008). Immune activation and inflammation in HIV-1 infection: causes and consequences. J Pathol..

[6] Balslev U, Monforte AD, Stergiou G, Antunes F, Mulcahy F, Pehrson PO (1997). Influence of age on rates of new AIDS-defining diseases and survival in 6546 AIDS patients. Scand J Infect Dis.

[7] Deeks SG, Phillips AN (2009). HIV infection, antiretroviral treatment, ageing, and non-AIDS related morbidity. BMJ.

[8] Grabar S, Weiss L, Costagliola D (2006). HIV infection in older patients in the HAART era. J Antimicrob Chemother.

[9] Nguyen N, Holodniy M (2008). HIV infection in the elderly. Clin Interv Aging.

[10] Nogueras M, Navarro G, Anton E, Sala M, Cervantes M, Amengual M, Segura F (2006). Epidemiological and clinical features, response to HAART and survival in HIV-infected patients diagnosed at the age of 50 or more. BMC Infect Dis.

[11] Bestilny LJ, Gill MJ, Mody CH, Riabowol KT (2000). Accelerated replication senescence of the peripheral immune system induced by HIV infection. AIDS..

[12] Grabar S, Kousignian I, Sobel A, Le Bras P, Gasnault J, Enel P (2004). Immunologic and clinical responses to highly active antiretroviral therapy over 50 years of age. Results from the French Hospital Database on HIV. AIDS..

[13] Yung RL, Mo R (2003). Aging is associated with increased human T-cell CC chemokine receptor gene expression. J Interferon Cytokine Res.

[14] Simone MJ, Appelbaum J (2008). HIV in older adults. Geriatrics..

[15] Giorgi JV, Hultin LE, McKeating JA, Johnson TD, Owens B, Jacobson LP (1999). Shorter survival in advanced HIV-1 infection is more closely associated with T lymphocyte activation than with plasma virus burden or virus coreceptor usage. J Infect Dis.

[16] Sousa AE, Carneiro J, Meier-Schellersheim M, Grossman Z, Victorino RM (2002). CD4 T-cell depletion is linked directly to immune activation in the pathogenesis of HIV-1 and HIV-2 but only indirectly to the viral load. J. Immunol..

[17] Deeks SG, Tracy R, Douek DC (2013). Systemic effects of inflammation on health during chronic HIV infection. Immunity.

[18] Ipp H, Zemlin AE, Erasmus RT, Glashoff RH (2014). Role of inflammation in HIV-1 disease progression and prognosis. Crit Rev Clin Lab Sci.

[19] Pratt G, Gascoyne K, Cunningham K, Tunbridge A (2010). Human immunodeficiency virus (HIV) in older people. Age Ageing.

[20] Utsuyama M, Hirokawa K, Kurashima C, Fukayama M, Inamatsu T, Suzuki K (1992). Differential age-change in the number of CD4+ CD45RA+ and CD4+ CD29+ T cell subsets in human peripheral blood. Mech Ageing Dev.

[21] Ferrando-Martinez S, Ruiz-Mateos E, Hernandez A, Gutierrez E, Rodriguez-Mendez Mdel M, Ordonez A, Leal M (2011). Age-related deregulation of naïve T cell homeostasis in elderly humans. Age (Dordr)..

[22] Perreau M, Savoye AL, De Crignis E, Corpataux JM, Cubas R, Haddad EK (2013). Follicular helper T cells serve as the major CD4 T cell compartment for HIV-1 infection, replication, and production. J Exp Med.

[23] Trickett A, Kwan YL (2003). T cell stimulation and expansion using anti-CD3/CD28 beads. J Immunol Methods.

[24] Papkalla A, Münch J, Otto C, Kirchhoff F (2002). Nef enhances human immunodeficiency virus type 1 infectivity and replication independently of viral coreceptor tropism. J Virol.

[25] Schindler M, Münch J, Kirchhoff F (2005). Human immunodeficiency virus type 1 inhibits DNA damage-triggered apoptosis by a Nef-independent mechanism. J Virol.

[26] Schindler M, Münch J, Kutsch O, Li H, Santiago ML, Bibollet-Ruche F (2006). Nef-mediated suppression of T cell activation was lost in a lentiviral lineage that gave rise to HIV-1. Cell.

[27] Wildum S, Schindler M, Münch J, Kirchhoff F (2006). Contribution of Vpu, Env, and Nef to CD4 down-modulation and resistance of human immunodeficiency virus type 1-infected T cells to superinfection. J Virol.

[28] Yu H, Khalid M, Heigele A, Schmökel J, Usmani SM, van der Merwe J, Münch J, Silvestri G, Kirchhoff F (2015). Lentiviral Nef proteins manipulate T cells in a subset-specific manner. J Virol.

[29] Munoz A, Sabin CA, Phillips AN (1997). The incubation period of AIDS. AIDS..

[30] Schnittman SM, Lane HC, Greenhouse J, Justement JS, Baseler M, Fauci AS (1990). Preferential infection of CD4+ memory T cells by human immunodeficiency virus type 1: evidence for a role in the selective T-cell functional defects observed in infected individuals. Proc Natl Acad Sci USA..

[31] Brenchley JM, Hill BJ, Ambrozak DR, Price DA, Guenaga FJ, Casazza JP (2004). T-cell subsets that harbor human immunodeficiency virus (HIV) in vivo: implications for HIV pathogenesis. J Virol.

[32] Gosselin A, Monteiro P, Chomont N, Diaz-Griffero F, Said EA, Fonseca S (2010). Peripheral blood CCR4+ CCR6+ and CXCR3+ CCR6+ CD4+ T cells are highly permissive to HIV-1 infection. J Immunol..

[33] Bryl E, Gazda M, Foerster J, Witkowski JM (2001). Age-related increase of frequency of a new, phenotypically distinct subpopulation of human peripheral blood T cells expressing lowered levels of CD4. Blood.

[34] Cané S, Ponnappan S, Ponnappan U (2012). Altered regulation of CXCR4 expression during aging contributes to increased CXCL12-dependent chemotactic migration of CD4(+) T cells. Aging Cell.

[35] Cortes MJ, Wong-Staal F, Lama J (2002). Cell surface CD4 interferes with the infectivity of HIV-1 particles released from T cells. J Biol Chem.

[36] Lama J, Mangasarian A, Trono D (1999). Cell-surface expression of CD4 reduces HIV-1 infectivity by blocking Env incorporation in a Nef- and Vpu-inhibitable manner. Curr Biol.

[37] Ross TM, Oran AE, Cullen BR (1999). Inhibition of HIV-1 progeny virion release by cell-surface CD4 is relieved by expression of the viral Nef protein. Curr Biol.

[38] Sallusto F, Lenig D, Mackay CR, Lanzavecchia A (1998). Flexible programs of chemokine receptor expression on human polarized T helper 1 and 2 lymphocytes. J Exp Med.

[39] Goodkin K, Shapshak P, Asthana D, Zheng W, Concha M, Wilkie FL (2004). Older age and plasma viral load in HIV-1 infection. AIDS..

[40] Sawhney M, Mathew M, Valarmathi MT, Das SN (2006). Age related changes in Fas (CD95) and Fas ligand gene expression and cytokine profiles in healthy Indians. Asian Pac J Allergy Immunol.

[41] Aggarwal S, Gupta S (1998). Increased apoptosis of T cell subsets in aging humans: altered expression of Fas (CD95), Fas ligand, Bcl-2, and Bax. J Immunol..

[42] Yalcin AD, Gorczynski RM, Kahraman MS, Demirel MU, Terzioglu E (2012). CD40, CD45 CTLA-4 levels are elevated in healthy older adults. Clin Lab..

[43] Riley JL, Schlienger K, Blair PJ, Carreno B, Craighead N, Kim D (2000). Modulation of susceptibility to HIV-1 infection by the cytotoxic T lymphocyte antigen 4 costimulatory molecule. J Exp Med.

[44] Day CL, Kaufmann DE, Kiepiela P, Brown JA, Moodley ES, Reddy S (2006). PD-1 expression on HIV-specific T cells is associated with T-cell exhaustion and disease progression. Nature.

[45] Kavathia N, Jain A, Walston J, Beamer BA, Fedarko NS (2009). Serum markers of apoptosis decrease with age and cancer stage. Aging (Albany NY)..

[46] Phelouzat MA, Laforge T, Arbogast A, Quadri RA, Boutet S, Proust JJ (1997). Susceptibility to apoptosis of T lymphocytes from elderly humans is associated with increased in vivo expression of functional Fas receptors. Mech Ageing Dev.

[47] Todo-Bom A, Mota-Pinto A, Alves V, Santos-Rosa M (2012). Aging and asthma – changes in CD45RA, CD29 and CD95 T cells subsets. Allergol Immunopathol (Madr)..

[48] Connor RI, Sheridan KE, Ceradini D, Choe S, Landau NR (1997). Change in coreceptor use correlates with disease progression in HIV-infected individuals. J Exp Med.

[49] Xiao L, Rudolph DL, Owen SM, Spira TJ, Lal RB (1998). Adaptation to promiscuous usage of CC and CXC-chemokine coreceptors in vivo correlates with HIV-1 disease progression. AIDS..

[50] Potts BJ, Maury W, Maring MA (1990). Replication of HIV-1 in primary monocyte cultures. Virology.

