# Supplementary material for: Increased susceptibility of CD4+ T cells from elderly individuals to HIV-1 infection and apoptosis is associated with reduced CD4 and enhanced CXCR4 and FAS surface expression levels
Source: Retrovirology. 2015 Oct 9;12:86. doi: 10.1186/s12977-015-0213-1 (PMC4600300; doi:10.1186/s12977-015-0213-1)
Supplement: Supplementary file 1 — 10.1186/s12936-015-0927-5 Increased levels of apoptosis in lymphocytes from elderly donors. Gating strategy of flow cytometry analysis of death and apoptosis rates from mock or HIV-1 NL4-3 reporter virus infected GFP+ or GFP− cells. Figure S2. Expression of activation markers and viral LTR activity in HIV-1 infected PBMC cultures from young and elderly individuals. (A, B) Representative primary data and statistical evaluations of the expression levels of (A) CD69 and (B) CD25 on X4 or R5 HIV-1 NL4-3 reporter virus infected GFP+ or GFP− cells from young (Y) and elderly (O) blood donors. (C) GFP expression levels in PBMC cultures from young and elderly donors infected with X4 or R5 HIV-1 NL4-3 reporter constructs. Each symbol represents the result obtained for one individual PBMC donor from the young (blue) or elderly (red) groups. [file 12977_2015_213_MOESM1_ESM.docx]

**Additional Files**

**Increased susceptibility of CD4+ T cells from elderly individuals to HIV-1 infection and apoptosis correlates with reduced CD4 and enhanced CXCR4 and FAS surface expression levels**

**Anke Heigele, Simone Joas, Kerstin Regensburger, Frank Kirchhoff**

**Two supplementary figures**


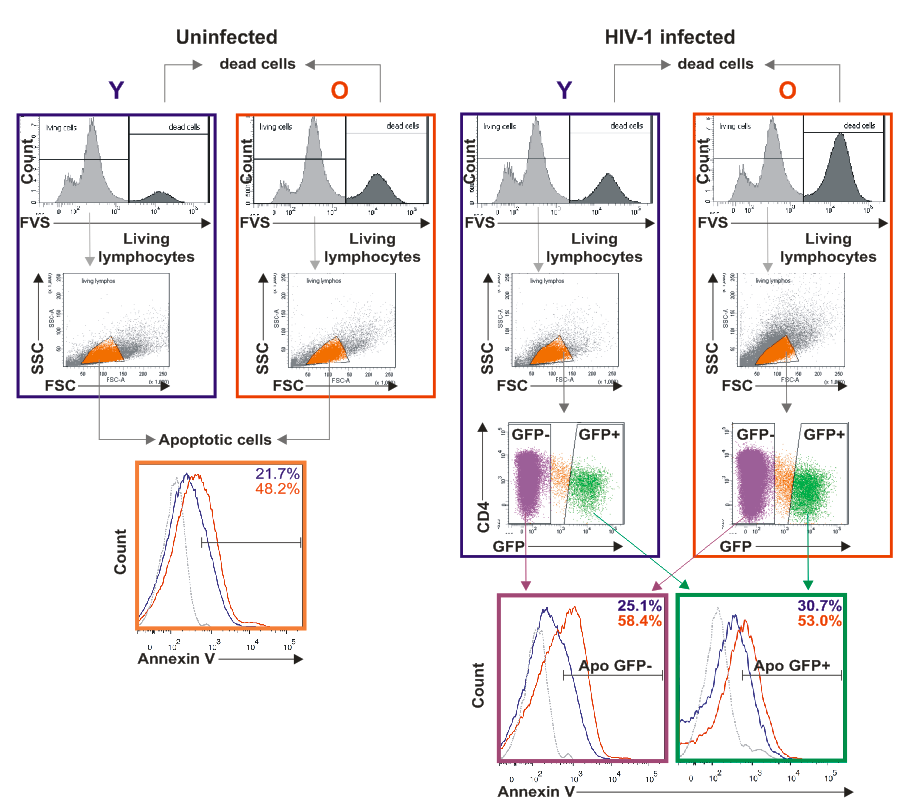


**Figure S1. Increased levels of apoptosis in lymphocytes from elderly donors.** Gating strategy of flow cytometry analysis of death and apoptosis rates from mock or HIV-1 NL4-3 reporter virus infected GFP+ or GFP- cells.


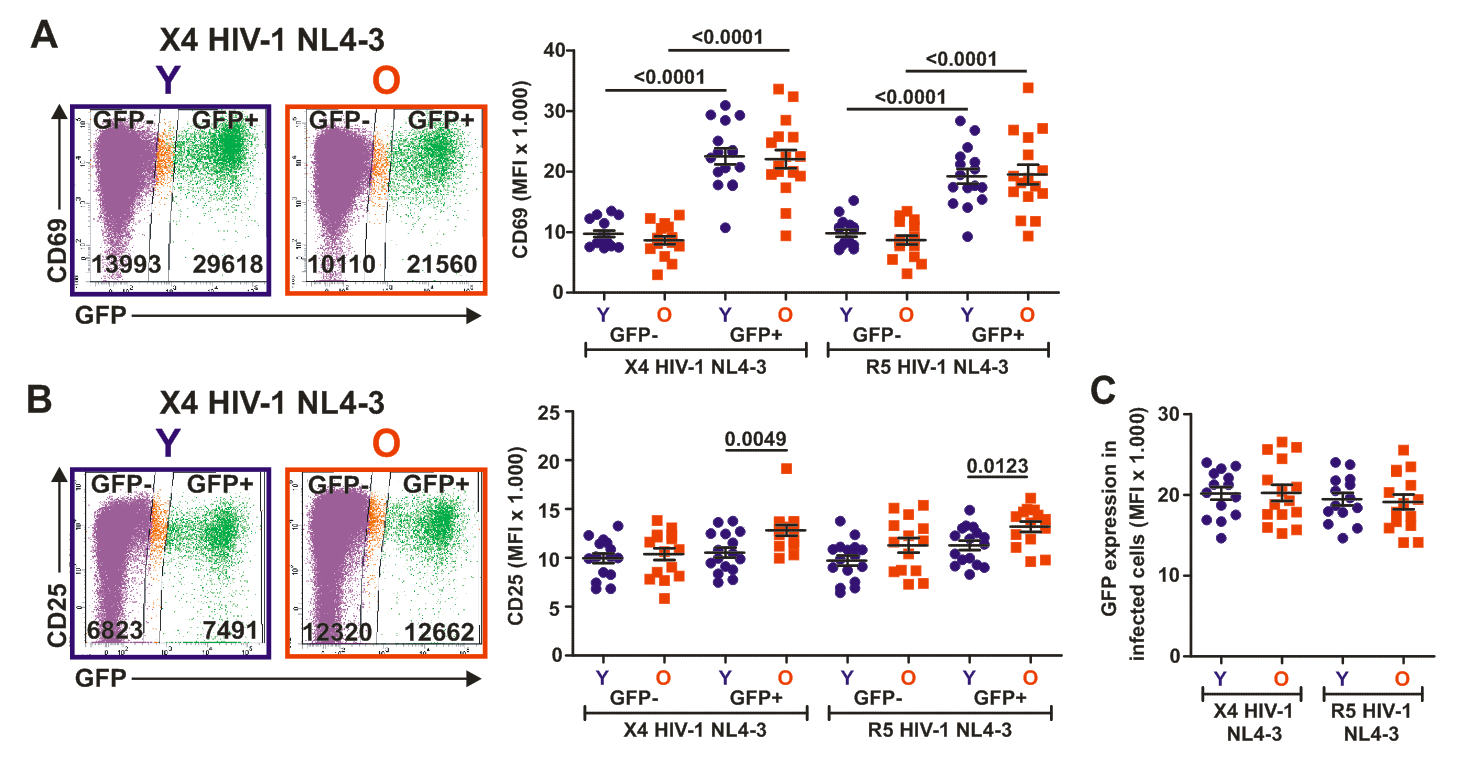
**Figure S2. Expression of activation markers and viral LTR activity in HIV-1 infected PBMC cultures from young and elderly individuals.** (A, B) Representative primary data and statistical evaluations of the expression levels of (A) CD69 and (B) CD25 on X4 or R5 HIV-1 NL4-3 reporter virus infected GFP+ or GFP- cells from young (Y) and elderly (O) blood donors. (C) eGFP expression levels in PBMC cultures from young and elderly donors infected with X4 or R5 HIV-1 NL4-3 reporter constructs. Each symbol represents the result obtained for one individual PBMC donor from the young (green) or elderly (red) groups.
